# Cerebral oxygen extraction fraction as an emerging imaging biomarker in mild cognitive impairment and dementia

**DOI:** 10.1590/1980-5764-DN-2025-0412

**Published:** 2026-09-14

**Authors:** Jawairya Muhammad Hussain, Aina Lashari, Wazeema Nageen, Amna Siddiqui, Abdul Waris, Syeda Maliha, Aisha Mudasir, Madyana Bibi Abdul Rehman, Mariyam Hifz ur Rahman

**Affiliations:** 1 Karachi Medical and Dental College, Karachi, Pakistan.; 2 Jinnah Sindh Medical University, Karachi, Pakistan.; 3 Liaquat College of Medicine and Dentistry, Karachi, Pakistan.; 4 Khyber Girls Medical College, Peshawar, Pakistan.

**Keywords:** Cerebrovascular Circulation, Alzheimer Disease, Dementia, Cognitive Dysfunction, Biomarkers, Neurovascular Coupling., Circulação Cerebrovascular, Doença de Alzheimer, Demência, Disfunção Cognitiva, Biomarcadores, Acoplamento Neurovascular.

## Abstract

The progressive onset of dementia and mild cognitive impairment (MCI) is a major global health concern due to its profound impact on quality of life. The challenge of identifying MCI and dementia lies partly in the high cost and invasiveness of some biomarkers. In this context, the oxygen extraction fraction (OEF) has emerged as a promising imaging biomarker. This review summarizes current evidence on cerebral OEF as a neuroimaging biomarker in MCI and dementia, focusing on pathophysiology, imaging techniques, comparison with established biomarkers, clinical applications, and future directions. A literature search was conducted from July 1–31, 2025, using the United States National Library of Medicine (PubMed), Scopus, and Web of Science. Search terms included “oxygen extraction fraction” OR “cerebral oxygen metabolism” AND (“mild cognitive impairment” OR “MCI” OR dementia OR “Alzheimer’s disease” OR “vascular dementia”) AND (qBOLD OR TRUST MRI OR mcQQ MRI OR 15O PET). English-language studies from the past ten years reporting OEF in cognitive impairment were included; case reports and non-human studies were excluded. OEF demonstrates distinct alterations across the spectrum of cognitive impairment. In MCI, reduced OEF has been linked to impaired neurovascular coupling and mitochondrial dysfunction. In dementia, patterns diverge — typically reduced in Alzheimer’s disease but heterogeneous in vascular conditions, often elevated under chronic hypoperfusion yet reduced when vascular pathology coexists with Alzheimer’s disease. Non-invasive techniques such as mcQQ magnetic resonance imaging (MRI) and TRUST MRI estimate OEF, while ^15^O PET remains the gold standard. OEF shows promise as a neuroimaging biomarker for early detection, differential classification, and monitoring disease progression in cognitive impairment.

## RESUMO

O início progressivo da demência e do comprometimento cognitivo leve (CCL) é uma importante preocupação de saúde global em razão do seu profundo impacto na qualidade de vida. O desafio de identificar o CCL e a demência reside, em parte, no alto custo e na invasividade de alguns biomarcadores. Nesse contexto, a fração de extração de oxigênio (OEF) surgiu como um biomarcador promissor de imagem. Esta revisão resume as evidências atuais sobre a OEF cerebral como biomarcador de neuroimagem no DCL e na demência, com foco na fisiopatologia, nas técnicas de imagem, na comparação com biomarcadores estabelecidos, nas aplicações clínicas e nas perspectivas futuras. A pesquisa bibliográfica foi realizada de 1º a 31 de julho de 2025, utilizando o United States National Library of Medicine (PubMed), o Scopus e o Web of Science. Os termos de busca incluíram “fração de extração de oxigênio” OU “metabolismo cerebral de oxigênio” E (“comprometimento cognitivo leve” OU MCI OU demência OU “doença de Alzheimer” OU “demência vascular”) E (qBOLD OU TRUST MRI OU mcQQ MRI OU 15O PET). Foram incluídos estudos em inglês dos últimos dez anos que relatassem a OEF no comprometimento cognitivo; relatos de casos e estudos em animais foram excluídos. A OEF apresenta alterações distintas ao longo do espectro do comprometimento cognitivo. No MCI, a redução do OEF tem sido associada ao comprometimento do acoplamento neurovascular e à disfunção mitocondrial. Na demência, os padrões divergem — tipicamente reduzido na doença de Alzheimer, mas heterogêneo em condições vasculares, frequentemente elevado sob hipoperfusão crônica, porém reduzido quando a patologia vascular coexiste com a doença de Alzheimer. Técnicas não invasivas, como a ressonância magnética mcQQ e a ressonância magnética TRUST, estimam o OEF, enquanto a ^15^O PET continua sendo o padrão-ouro. A OEF mostra-se promissora como biomarcador de neuroimagem para a detecção precoce, classificação diferencial e monitoramento da progressão da doença no comprometimento cognitivo.

## INTRODUCTION

**D**ementia is a common disorder characterized by cognitive impairment (CI) affecting daily life^1^. Mild cognitive impairment (MCI) describes individuals transitioning between normal aging and dementia^2^. A 2016 US study found 33.33% of 3,496 adults aged 65+ had dementia or MCI, highlighting age as a major risk factor^1^. In 2017, 10% of Americans over 65 had significant cognitive issues^1^. A 2019 US study reported 9.7% dementia prevalence among 54.1 million people aged 65+, rising to 11.3% by 2020^1^. A 2021 US cohort also showed 11.3% dementia prevalence in those 65+^1^. Globally, MCI prevalence was 19.7% in 2023, with higher rates reported in hospitals post-COVID-19^2^.

A recent study reported a 15.4% prevalence of MCI in China, linked to sedentary habits (alcohol intake, inactivity) and comorbidities such as metabolic, cardiac, and psychological disorders^2^. Low estrogen levels with aging increase susceptibility in older women, explaining their higher risk of MCI and dementia compared with men^3^. Dementia prevalence is also greater in rural areas and among individuals with parental history^4^. Early diagnosis remains difficult due to the lack of biomarkers, limited cognitive assessment tools, and clinical time constraints^3^. Furthermore, disclosure of risk often causes psychological distress, complicating patient acceptance^5^.

Early detection of MCI or dementia enables timely intervention, which may slow disease progression and mitigate further cognitive decline^6^. Although cerebrospinal fluid (CSF) biomarkers such as the Aβ42/Aβ40 ratio, total tau (t-tau), and phosphorylated tau (p-tau) are well established for the diagnosis of MCI and Alzheimer’s disease (AD), their widespread clinical implementation remains limited by invasiveness, cost, and assay-dependent variability in cut-off values, which hinders cross-center standardization^7^. The use of CSF biomarkers and amyloid positron emission tomography (PET) is not easily accessible, especially for low-income individuals, and can also lead to side effects such as headaches due to the invasive procedure, particularly after a lumbar puncture for detecting CSF biomarkers^8^. Another biomarker that can be used for MCI or dementia is the oxygen extraction fraction (OEF), which estimates oxygen usage by the body’s tissues^9^.

The cerebral OEF quantifies the brain’s capacity to extract oxygen from arterial blood, with a standard value around 40%, varying according to metabolic demand^9^. This review summarizes current evidence on OEF as a neuroimaging marker in early cognitive decline, covering its pathophysiological basis, imaging methods such as magnetic resonance imaging (MRI) and PET, comparisons with established and emerging biomarkers, clinical relevance, translational potential, limitations, and future directions.

Throughout this review, we distinguish between vascular dementia — the clinical syndrome resulting from cerebrovascular disease — and the broader category of vascular pathology, which includes large-artery atherosclerosis, cerebral small vessel disease (CSVD), cerebral amyloid angiopathy (CAA), and chronic hypoperfusion. These distinct entities have different effects on cerebral oxygen metabolism and OEF, as discussed in subsequent sections.

## PATHOPHYSIOLOGICAL BASIS OF OXYGEN EXTRACTION FRACTION ALTERATION IN COGNITIVE DECLINE

### Brain energy metabolism: oxygen extraction fraction as a marker of cerebral metabolic demand vs. supply

Cerebral OEF is a measure of how much oxygen is taken up from blood as it is flowing through the capillaries and is used to determine the oxygen demand and supply in the human brain. It is an important biomarker and is essential in studying pathophysiology for a variety of diseases like stroke, AD etc. Hence, measurement of OEF holds key value while diagnosing and treating neurological conditions, as it helps determine its activity^10^.

### Neurovascular coupling and its dysfunction in mild cognitive impairment and Alzheimer’s disease

Under normal conditions, cerebral oxygen supply matches demand, but disease disrupts this balance^11^. Neurovascular coupling (NVC), the mechanism linking neuronal activity to cerebral blood flow (CBF), is impaired in cortical and subcortical regions in MCI and AD^12^. One study showed reduced activity in the dorsolateral prefrontal cortex (DLPFC) of MCI patients compared with cognitively normal individuals, measured by regression coefficients (β) from a general linear model of brain connectivity (F3-F5, F3-F1, F3-FC3).

NVC responses were assessed using functional near-infrared spectroscopy (fNIRS) by measuring continuous changes in cerebrocortical hemodynamic signals during an n-back working memory paradigm. White matter hyperintensities (WMH), representing ischemic lesions, were assessed using 1.5-Tesla T2-weighted fluid-attenuated inversion recovery (FLAIR) MRI and quantified using the Fazekas scale. To examine task-related functional connectivity, normalized node degree was calculated for each brain area, which represents the functional connectivity of a brain area with other areas during a cognitive activity. Normalized node degree quantifies the number of connections (edges) a node has relative to the total possible connections in the network, yielding a standardized measure of functional integration during the cognitive task. In the MCI group (n=18), the median NVC response was 1.401 (interquartile range — IQR: −17.27 to 14.16), whereas the age-matched controls Cognitively Normal (CN) group (n=19) demonstrated a markedly higher median of 22.86 (IQR: 9.180 to 37.30), representing a statistically significant difference (p=0.0033). Connectivity of the DLPFC with other cortical regions, quantified by DLDP¯, was consistently lower in MCI participants across cognitive tasks (95% confidence interval [CI] for the difference: 0.0254–0.1273, p=0.0036).

During the n-back working memory task, the participants were presented with tasks of varying levels of difficulty (e.g., 0-back, 1-back, 2-back). This included the 0-back task, which was the baseline task. In block-design n-back paradigms, each level of the task is presented repeatedly in multiple blocks to assess performance and brain activation patterns, with the 0-back condition serving as the baseline control (participants respond to predefined targets). Results showed that the MCI group (n=20) had significantly low normalized node degree values compared to the cognitively normal controls (n=18), as revealed during the second block of the 0-back task (CI: 0.0326–0.2963, p=0.0078). This indicated that the functional connectivity was compromised in the MCI group. Thus, it was concluded that MCI was linked to alterations in the network connectivity of the cortex, which could be captured using the fNIRS technique. Task-averaged normalized node degree values also trended lower in MCI (0.7590±0.1650) relative to CN (0.8429±0.1062), a difference that did not reach statistical significance (p=0.0727) but was supported by a moderate effect size (Cohen’s D=0.6047)^12^.

### Hypoperfusion, microvascular dysfunction, and compensatory oxygen extraction fraction changes

AD is characterized by amyloid plaques and tau tangles, but many patients also develop cerebral amyloid angiopathy (CAA), with amyloid deposition in cerebral vessels. Post-mortem studies report moderate to severe CAA in up to 98% of AD cases, associated with faster cognitive decline, whereas MRI biomarkers such as lobar microbleeds detect it in only ~22% of living patients. Cerebrovascular Aβ deposition in CAA triggers early microvascular dysfunction — including stiffened vessel walls, impaired neurovascular regulation, disrupted blood brain barrier (BBB), and chronic cerebral hypoperfusion — which precedes hemorrhagic lesions. These cerebrovascular changes contribute independently to dementia progression beyond classical AD pathology^13^. Impaired NVC during visual stimulation, as measured by task-evoked cerebrocortical hemodynamic responses using fNIRS, serves as a sensitive marker of CAA burden, with sporadic CAA showing progressive decline and Dutch-type hereditary CAA exhibiting presymptomatic deficits. Longitudinal data confirm worsening NVC over four years, independent of hemorrhagic events, underscoring its role as an early indicator of cerebrovascular dysfunction in AD and CAA^13^. In patients with chronic small-vessel disease, reduced CBF prompts compensatory OEF elevation to preserve cerebral metabolic rate of oxygen (CMRO₂)^11^. PET studies in cognitively normal individuals report resting OEF of 0.35±0.06 in gray matter and 0.39±0.06 in the whole brain. When OEF compensation fails, CMRO₂ falls, raising ischemic injury risk. The lack of oxygen, if not addressed, results in dysfunction and damage, and small vessel disease and Aβ further increase vulnerability^11^.

The two-hit vascular hypothesis proposes that genetic, vascular, and environmental risks contribute to neuronal injury through an Aβ-independent pathway (hit 1: vascular injury) and an Aβ-dependent pathway (hit 2). These may act separately or synergistically to drive neurodegeneration. The Aβ-independent route involves BBB breakdown, capillary narrowing, and reduced CBF, leading to hypoxia and toxic metabolite accumulation, which in turn promote Aβ deposition. Both reduced CBF and Aβ accumulation contribute to tau hyperphosphorylation and neuroinflammation.

In addition to the two-hit vascular hypothesis, the capillary dysfunction hypothesis emphasizes impaired oxygen extraction (OE) from uneven capillary blood flow, quantified as capillary transit time heterogeneity (CTH). High CTH reduces oxygen uptake by creating “shunts” of oxygen-rich blood. Normally, CTH decreases to meet oxygen demand, but in AD this mechanism fails, impairing oxygen delivery. Initially, elevated CTH can be offset by increasing CBF, but chronic worsening eventually lowers tissue oxygenation, fostering Aβ buildup, inflammation, and BBB injury. This model shifts the focus from reduced CBF to inefficient capillary oxygen exchange as the earliest vascular trigger in AD^14^. Mitochondrial dysfunction and impaired oxidative phosphorylation further exacerbate amyloid toxicity and glial inflammation, driving synaptic loss and CI.

### Relationship between oxygen extraction fraction, oxidative stress, and mitochondrial dysfunction in early neurodegeneration

**Figure 1:**
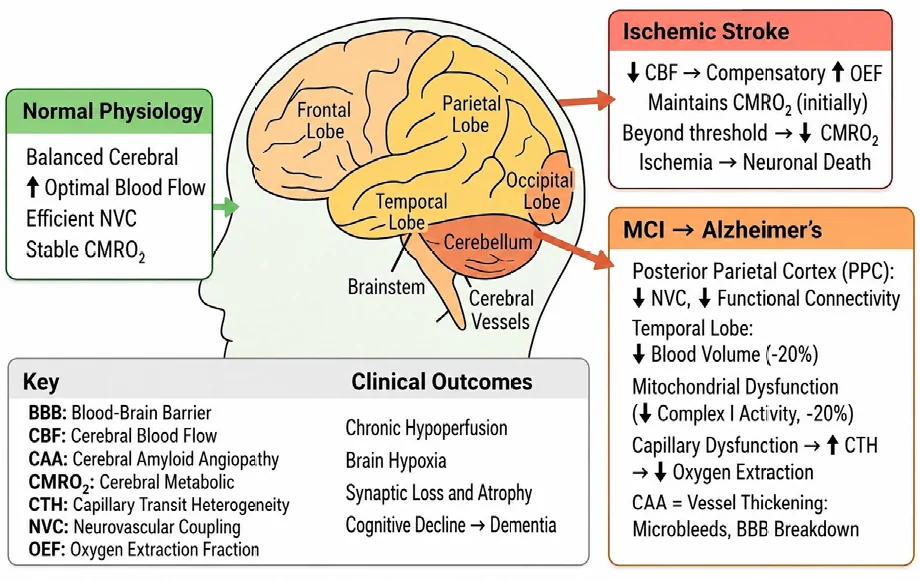
The figure contrasts normal brain oxygen use with mild cognitive impairment (MCI) and Alzheimer’s pathways leading to hypoxia and dementia.

Abbreviations: CAA, Cerebral amyloid angiopathy; CTH, Capillary transit time heterogeneity; BBB, Blood Brain Barreir.

A recent study found that decreases in mitochondrial activity and brain volume are the most sensitive signs of AD progression. At diagnosis, mitochondrial complex I activity was already reduced by 28% in the caudate, 25% in the hippocampus, and 23% in the thalamus as measured *in vivo* using [^18F] BCPP-EF PET imaging of complex I binding in early AD patients^15^. The frontal and dorsolateral prefrontal cortices were also 12% lower compared to healthy people. One year later, activity dropped further by 11% in the parietal lobe, 10% in the precuneus, and 12% in the DLPFC. For brain volume, the hippocampus was 23% smaller and the temporal lobe 16% smaller at diagnosis, with an additional 5 and 4% shrinkage in the following year. Importantly, these changes occurred independently of CBF, showing that mitochondrial impairment is not simply due to perfusion loss. In summary, early AD is marked by reduced functional reserve, especially in oxidative metabolism and synaptic integrity. If these changes are directly linked to cognitive decline, they could serve as important treatment targets and be tracked with imaging biomarkers^15^. Figure 1 illustrates the contrast between normal cerebral oxygen utilization and the altered pathways observed in MCI and AD.

## IMAGING TECHNIQUES TO MEASURE OXYGEN EXTRACTION FRACTION

Early identification of MCI and dementia has become increasingly crucial. Advances in imaging, particularly MRI and PET-based techniques, now allow more precise assessment of OEF, offering valuable insights into disease detection and progression.

### Magnetic resonance imaging-based methods

#### Quantitative Blood Oxygen Level-Dependent (qBOLD)

Quantitative blood oxygen level-dependent (qBOLD) MRI uses multi-echo gradient-echo scans to simulate magnetic field changes caused by deoxyhemoglobin in venous blood, measuring parameters such as deoxygenated blood volume (DBV) and reversible transverse relaxation (R2′). These parameters are used in biophysical signal models to compute the OEF, linking BOLD signal decay to deoxyhemoglobin concentration in venous blood. Because the measurements are taken from voxel-wise MRI signal modeling, qBOLD may produce spatially defined maps of OEF, allowing for the study of regional changes in oxygen metabolism rather than just overall brain values^16^. Importantly, qBOLD acquisitions are typically performed at rest and do not require gas challenges, making the technique suitable for both clinical and research applications. qBOLD has differentiated aggressive breast tumors by identifying low oxygen and new blood vessels without invasive tests^16^. Repeated qBOLD scans showed consistent OEF values of 28–38% under hypercapnia, closely matching dual-gas BOLD (37vs.39%). It demonstrated higher reliability for OEF than DBV (variability 3.3 vs. 11%), detecting significant changes with as few as five subjects at 80% power^17^. In dementia, high-resolution qBOLD revealed distinct OEF patterns across disease stages, notably in fronto-parietal and temporal regions^18^.

#### T^2^-Relaxation-Under-Spin-Tagging Magnetic Resonance Imaging (TRUST MRI)

T₂-Relaxation-Under-Spin-Tagging (TRUST) MRI estimates venous blood oxygenation by isolating the MRI signal of venous blood and measuring its transverse relaxation time (T₂b). Because blood T₂ relaxation varies with hemoglobin oxygen saturation level, this measurement can be converted into venous oxygenation, which is then used together with arterial oxygen saturation to calculate global cerebral OEF. TRUST is fast, reliable, and delivers high-quality data^19^. TRUST typically acquires superior sagittal sinus venous signals in a brief acquisition (often completed in under 5 minutes, with optimized protocols taking as little as 1.2 minutes^20^) and estimates whole-brain OEF by measuring blood T₂ relaxation, from which venous oxygen saturation is derived. A 2023 study confirmed its accuracy during controlled gas challenges, showing that TRUST could pick up physiological changes and match more complex methods^19^. TRUST-derived OEF values have shown strong agreement with T₂-Transverse Relaxation Interleaved Refocusing (T₂-TRIR), an MRI technique that measures blood T₂ relaxation through interleaved refocusing pulses to estimate venous oxygenation, in various respiratory conditions. This agreement was supported by statistical analyses such as regression and Bland-Altman comparisons, with significance confirmed at the 0.05 level^19^. In a separate validation study, TRUST MRI estimated a global OEF of 36.44 ± 4.07%, closely matching the 36.45 ± 3.65% measured by ^15O PET in 16 healthy adults.^20^ Although TRUST traditionally provides global OEF measurements, recent developments in sequence design and machine-learning-based reconstruction are being explored to improve regional assessment of cerebral oxygen metabolism^20^.

#### Quantitative Susceptibility Mapping (QSM)-based approaches

The paramagnetic characteristics of deoxyhemoglobin are used by magnetic susceptibility-based MRI methods to measure cerebral oxygenation. Susceptibility-weighted imaging (SWI) and phase imaging are examples of early susceptibility-sensitive techniques that mainly improve venous structure visualization and offer qualitative data regarding cerebral vasculature. However, these approaches do not provide reliable quantitative measurements of OEF. In contrast, quantitative susceptibility mapping (QSM) enables quantitative estimation of magnetic susceptibility differences caused by deoxyhemoglobin in venous blood, which can be incorporated into biophysical models to estimate regional OEF^20^.

A more advanced approach integrates QSM with qBOLD modeling to jointly estimate venous susceptibility and blood oxygenation from the same multi-echo gradient-echo acquisition. This combined framework, often referred to as multi-echo complex quantitative susceptibility mapping plus qBOLD (mcQQ) MRI methods (QSM+qBOLD), improves OEF estimation by simultaneously modeling magnetic susceptibility and BOLD signal decay, reducing uncertainty compared with using either technique alone^21^.

In AD, QSM combined with qBOLD (QQ) detected reductions in OEF and CMRO₂ in regions such as the hippocampus and parieto-temporal cortex, findings that correlated with cognitive decline^18^. QSM-OEF showed good agreement with PET, with sensitivity up to 82% and specificity up to 89% in detecting elevated OEF in cerebrovascular patients^22^. This approach uses multi-echo complex MRI data to jointly reconstruct susceptibility maps and BOLD parameters. In this framework, multi-echo complex QSM (mcQSM) refers to the susceptibility reconstruction component, while mcQQ denotes the integrated approach that combines mcQSM with qBOLD modeling to estimate OEF. By using both magnitude and phase information from a single multi-echo acquisition, mcQQ improves the stability and accuracy of regional OEF estimation. This approach has demonstrated improved performance in detecting low-OEF regions in both simulations and stroke patients^21^.

In addition to mcQQ, more advanced algorithmic variants such as quantitative susceptibility mapping plus quantitative blood oxygen level-dependent with temporal clustering, tissue composition, and total variation regularization (QQ-CCTV) — which incorporates temporal clustering, tissue composition, and total variation regularization — have been proposed to improve OEF map accuracy; these are discussed in Section 6. Other MRI techniques, such as dynamic susceptibility contrast (DSC)-MRI, use rapid T2-weighted imaging during the first pass of a gadolinium-based contrast bolus to generate perfusion maps of CBF, CBV, and mean transit time (MTT). These perfusion parameters, which can inform OEF interpretation in the context of ischemia and vascular dysfunction, are discussed later in Section 4.5 in relation to cognitive impairment^23^.

#### Limitations

Although MRI-based methods avoid radiation and are easier to access than PET, they come with some challenges:

- qBOLD MRI needs complex signal modeling and is sensitive to magnetic field issues, which can affect accuracy^16^.
- TRUST MRI primarily provides global cerebral OEF measurements and has limited ability to resolve regional variations in oxygen metabolism^20^.
- QSM-based approaches can be affected by noise, susceptibility artifacts, and assumptions used in OEF modeling^21^.
- mcQQ MRI offers better detail but needs complicated scans and long processing, so it’s still used only in research^21^.

### Positron emission tomography (PET)-based approaches

#### ^15^O PET Techniques

Triple-oxygen PET using ^15^O-labeled H₂O, ^15^O-O_2_, and ^15^O-CO is considered the gold standard for measuring cerebral oxygen metabolism. Through separate inhalation protocols and kinetic modeling, this approach enables quantitative assessment of CBF, OEF, and the CMRO_2_, providing precise regional measurements of brain oxygen metabolism^22^. This technique provides highly accurate quantitative measurements, and has been extensively validated in clinical and research settings. Serial scans using inhalation protocols can evaluate multiple cerebral parameters like blood flow and metabolism^24^. Although traditional ^15^O PET requires arterial sampling for input function modelling, new PET/MRI approaches now allow image-derived input functions, making the process less invasive^25^. PET shows high specificity but moderate sensitivity for detecting abnormal cerebral blood volume, with correlation values ranging from R^2^=0.47 to 0.58 when compared to MRI^22^. A PET-only study using ^15O-water showed high accuracy for gray matter CBF, with a strong correlation (R^2^=0.92) and just 2.1% bias — better than other PET/MRI methods^25^.

#### Limitations

Despite its strengths, ^15^O PET has several drawbacks:

- Short 2-minute half-life demands on-site cyclotron, restricting access^20^.
- Inhalation makes results sensitive to mask fit and breathing patterns^24^.
- Short scan frames cause noisy images, reducing voxel-level accuracy^24^.
- Arrival time mapping needs over 20 minutes of processing — too long for routine use^24^.
- Ionizing radiation exposure restricts repeated scans and use in at-risk individuals^22^.

Table 1 summarizes major OEF imaging techniques across key parameters.

**Table 1:** Comparison of OEF Imaging Modalities: Technical Principles and Clinical Applicability.

Imaging Modality	OEF Estimation Method	Quantitative Parameters	Advantages	Limitations	Clinical use stage
qBOLD MRI	Models MR signal dephasing due to deoxyhemoglobin; estimates OEF using R__2_′_ and DBV	OEF, DBV, SvO__2__	Non-invasive; regional OEF mapping; reproducible measurements^16,18^	Field inhomogeneity; complex modeling; spatial limits	Research use; early clinical trials^16,17^
TRUST MRI	T__2__ relaxation of venous blood; calculates OEF from venous oxygenation	OEF (global), SvO__2__	Fast, reproducible; no radiation or contrast; detects physiological change^19,20^	Limited to large vessels; low spatial resolution	Research use; not routine clinically^19^
SWI/QSM MRI	Magnetic susceptibility of deoxyhemoglobin; SWI visualizes veins qualitatively, while QSM enables quantitative susceptibility estimation for OEF modeling.	Magnetic susceptibility, venous oxygenation (model-derived OEF)	High spatial detail; non-invasive; shows small veins^18,20,21^	Semi-quantitative; sensitive to artifacts^20,21^	SWI is clinical; QSM in validation^21^
mcQQ MRI	Combines mcQSM with qBOLD in multi-echo GRE scan	OEF, venous susceptibility, T2 decay	Better accuracy via dual contrast; high resolution^21^	Complex modeling; needs simultaneous acquisition	Experimental/research only^21^
^15^O PET	Inhaled ^15O tracers with kinetic modeling; arterial or image-derived input	CBF, OEF, CMRO__2__	Precise, quantitative measurement of CBF, OEF and CMRO__2__; gold standard^22,24^	Requires cyclotron; radiation; patient prep; sampling^22,24,25^	Research/PET-MRI centers^22^

Abbreviations: OEF, oxygen extraction fraction; DBV, deoxygenated blood volume; SvO_2_, venous oxygen saturation; CBF, cerebral blood flow; CMRO_2_, cerebral metabolic rate of oxygen; QSM, quantitative susceptibility mapping; qBOLD, quantitative blood oxygen level-dependent; mcQQ, multi-echo complex QSM + qBOLD; GRE, gradient echo; PET, positron emission tomography; SWI, Susceptibility-Weighted Imaging.

## EVIDENCE OF OXYGEN EXTRACTION FRACTION CHANGES IN MILD COGNITIVE IMPAIRMENT AND EARLY DEMENTIA

### Oxygen extraction fraction variation with aging across the cognitive impairment spectrum

An MRI study using QSM and qBOLD imaging found that OEF declines with age in cortical gray matter and whole brain, but not in white matter, among cognitively intact older adults. Age-related OEF reduction was observed regionally in the superior frontal gyri, hippocampi, and fusiform gyri after correction. Aging is a known risk factor for dementia, associated with increased Aβ deposition and cellular stress. Since OEF reflects brain metabolism through oxygen utilization, declining OEF with age may indicate impaired neuronal function and metabolic inefficiency.

In contrast, OEF appeared to be less sensitive to age-related deterioration in people with CI, suggesting that once CI is established, disease-related effects on brain oxygen use, such as the damaging effects of P-tau and Aβ plaques on mitochondria, may outweigh age-related consequences. Nevertheless, in cognitively impaired individuals, lower OEF was linked to higher white matter hyperintensities load in areas such as the caudate, hippocampus, superior temporal gyri, and white matter nuclei, indicating that certain forms of vascular damage may be linked to reduced rather than increased oxygen extraction^26^. White matter hyperintensities are widely recognized as imaging markers of chronic cerebral small-vessel disease (CSVD) and represent an important pathological substrate of vascular dementia^27^. In cognitively impaired individuals, these vascular changes may coexist with underlying AD pathology and may further contribute to reduced OEF, indicating impaired brain metabolism. This is consistent with the two-hit hypothesis of AD, which states that CSVD and preexisting AD pathology work together to significantly reduce OEF, more so than either pathology would on its own^26^.

### Alzheimer’s disease vs. vascular dementia patterns

AD and vascular cognitive impairment and dementia (VCID) are common and often coexist. AD lowers neural activity due to amyloid deposition, tau tangles, and neuronal loss, reducing glucose and oxygen use and thus lowering OEF despite preserved blood flow. Lower OEF correlates with higher amyloid burden (β=92.12±41.23, p=0.03), indicating OEF reflects AD pathology^28^. Research identified hevin, an astrocyte protein vital for synapse maintenance, as a therapeutic target; restoring hevin in AD models improves memory without affecting amyloid plaques, suggesting new intervention pathways beyond amyloid clearance.

Cerebral hypoperfusion due to primary vascular insufficiency — such as chronic carotid occlusion or small vessel cerebrovascular disease — can elicit compensatory increases in OEF, reflecting the brain’s attempt to preserve metabolic demands in the face of reduced blood flow^9^. In contrast, primary neurodegenerative disorders such as AD are typically characterized by reduced neural metabolism and diminished OEF^28^. A prospective TRUST-MRI study showed OEF rose significantly in high vascular risk older adults, correlating with white matter lesion progression but not AD markers^9^. In low-risk subjects, OEF increased minimally. Another TRUST-MRI study found OEF higher in individuals with vascular risk (β=1.36±0.55, p=0.02), but lower in dementia and mild cognitive impairment (β=–2.70±1.15, p=0.02), reflecting reduced oxygen extraction with cognitive decline. In low-risk people, higher OEF linked to better processing speed^28^.

Overall, OEF patterns differ by pathology. In AD, OEF decreases due to metabolic decline and amyloid burden. In vascular cognitive impairment, OEF responses are heterogeneous: it often rises as a compensatory mechanism in chronic hypoperfusion states (e.g., carotid stenosis or early small vessel disease), but may ultimately decline in advanced small vessel disease, where extensive white matter damage impairs metabolic capacity. These divergent patterns suggest that OEF could help differentiate AD from vascular etiologies, detect early MCI in at-risk populations, and identify early dysfunction in genetically predisposed individuals such as APOE4 carriers^28^.

### Influence of Apolipoprotein E genotype

An MRI/TRUST MRI study in older adults found that Apolipoprotein E (APOE-ε4) carriers, at higher AD risk, and those with cognitive impairment showed poorer brain health, marked by lower CMRO_2_ and, to a lesser extent, lower OEF. Strong correlations existed between CMRO_2_ and structural brain integrity and cognitive function. Higher CMRO_2_ associated with better performance on the Boston Naming Test (β=0.05, 95%CI = 0.01–0.08, p=0.004), Episodic Memory Composite (β=0.01, 95%CI = 0.004–0.02, p=0.003), Executive Function Composite (β=0.01, 95%CI=0.003–0.02, p=0.01), and Hooper Visual Organization Test (β=0.05, 95%CI=0.02–0.08, p=0.004), with significance after false discovery rate correction. No significant OEF-cognition correlation was seen in APOE-ε4 carriers regardless of cognitive status. However, in APOE-ε4 homozygotes, lower OEF linked to smaller hippocampal volume (β=26, 95%CI=6–46, p=0.01) and poorer memory (β=0.03, 95%CI=0.009–0.05, p=0.005). These results suggest CMRO_2_ as a more robust biomarker than OEF, especially when combined with APOE-ε4 allele count and status, while OEF remains useful in detecting region-specific cerebrovascular changes in high-risk groups like APOE-ε4 homozygotes^29^.

### Role of vascular risk and small vessel disease

According to a PET-MRI carried out on older adults with a range of vascular disease risk and varied cognitive function, higher OEF may make up for lower CBF, preserving oxygen delivery and safeguarding cognitive performance. Every region of the brain showed a significant inverse connection between CBF and OEF, with regional variations consistent with other research. Additionally, higher OEF was also associated with longer arterial transit times (ATT) in individuals with higher vascular risk and CI, which may be the result of longer microvascular routes. Research suggests that OEF increases with vascular risk and WMH progression, indicating its potential as a marker for small vessel disease. Increased OEF may be a temporary compensatory response, but sustained increases, especially when vascular burden is severe, may indicate a move toward cerebrovascular insufficiency and cognitive risk^30^.

### Association with cognitive testing and brain volumes

A dynamic susceptibility contrast (DSC)-MRI study in cognitively impaired adults found elevated OEF linked to aging and smoking, indicating cerebrovascular stress. White matter hyperintensities volume was significantly higher and CMRO_2_ lower in vascular dementia than in subjective cognitive impairment (SCI), MCI, and AD. CMRO_2_ decreased progressively from SCI (13.1±3.0 μmol/100 g/min), MCI (12.9±3.0), and AD (12.0±2.4) to vascular dementia (9.3±2.2, p=0.001), suggesting its role in distinguishing vascular dementia. Although OEF was slightly higher in AD (0.48±0.07) and vascular dementia (0.47±0.09) versus SCI (0.44±0.06), differences were not statistically significant (p=0.117). Cognitive performance inversely correlated with white matter hyperintensities volume and OEF, both negatively associated with Mini-Mental State Examination scores, unlike CMRO_2_. OEF was similar across dementia types, whereas CMRO_2_ was lowest in vascular dementia, indicating OEF reflects global metabolic stress and CMRO_2_ aids subtype differentiation^31^. Table 2 summarizes clinical studies applying OEF in MCI and dementia, highlighting population differences and findings.

**Table 2:** Summary of clinical studies using OEF in MCI/dementia.

Study Year	Population Studied	OEF imaging method used	Key Findings	Brain Regions Studied	Cognitive/ Biomarker Correlation
Chiang et al. (2022)^26^	Older adults; cognitively impaired and cognitively intact.	MRI combining QSM and BOLD	OEF declines with age in cognitively intact. In cognitively impaired no significant change of age with OEF; lower OEF was linked to higher WMH load	Cortical gray matter; frontal, parietal, temporal cortex, precuneus, posterior and isthmus cingulate, hippocampus, caudate, white matter regions	Cognitively intact: aging → ↓ OEF → reflect age-related metabolic decline. Cognitively impaired: OEF less sensitive to age→disease pathology dominate aging effects + ↑ WMH burden → ↓OEF → reflects vascular-related metabolic dysfunction + AD
Lin et al. (2022; published Feb 2023)^9^	Older adult population with developing vascular and AD pathology	TRUST-MRI	↑ OEF in high vascular risk. OEF change linked to vascular risk + WMH volume. No association with CSF biomarkers of AD.	Global measure (via superior sagittal sinus)	↑ OEF⟶↑ vascular risk and WMH growth→ reflects vascular disease progression. OEF changes independent of AD pathology.
Jiang et al. (2020)^28^	Individuals with MCI, dementia, and cognitively normal subjects.	TRUST-MRI	AD; ↓OEF → poor cognition higher amyloid. VCI; higher OEF →↑ vascular risk. OEF linked to better cognition in those with low vascular risk. Higher vascular risk scores → ↑ OEF	Global measure (via superior sagittal sinus)	AD: Low OEF = worse cognition + more amyloid → reflects neuronal or metabolic failure VCI: High OEF = high vascular risk → reflects vascular compensation. Low vascular risk associated with better cognition
Robb WH et al. (2022)^29^	Cognitively impaired and unimpaired older adults	MRI/TRUST MRI	No main effect of OEF on cognition. APOE-ε4 carriers →↓CMRO_2_ link to poor cognition. In homozygous APOE-ε4, ↓OEF is related with ↓ hippocampal volume + impaired memory function.	Hippocampus and inferior lateral ventricles, broader gray matter volume	APOE-ε4 carriers →↓CMRO_2_ → poorer language and episodic memory. OEF showed modest associations with episodic memory and hippocampal volume only in relation to APOE-ε4 allele count; no strong independent effect on cognition.
McFadden J et al. (2025)^30^	Older adults with a range of vascular disease risk and varied cognitive function	PET–MRI	↑OEF helps sustain cognition when ↓ CBF. ↑OEF→ longer ATT in those with vascular risk and cognitive decline.	Venous drainage territories	↑ATT → cognitive impairment. ↑ OEF with ↑vascular risk and ↑ATT. No regional specificity: OEF changes reflect global cerebrovascular compensation.
Lee et al. (2025)^31^	Cognitively impaired adults (includes MCI, vascular dementia, AD, history of infarct)	DSC-MRI	Smoking/older age → ↑ OEF. ↑ WMH volume + ↓CMRO_2_→ vascular dementia. Cognitive decline →negative correlation with WMH volume + OEF. OEF +WMH volume show inverse relation with MMSE scores.	White matter hyperintensity (WMH) regions across the brain	↑OEF and ↑ WMH volume were negatively correlated with global cognition (MMSE). ↓ CMRO_2_ in vascular dementia compared to other → vascular injury marker. OEF across dementia types → No significant variation → not specific to AD or VD

Abbreviations: OEF, oxygen extraction fraction; MRI, Magnetic Resonance Imaging; QSM, Quantitative Susceptibility Mapping;

BOLD, Blood Oxygen Level-Dependent; WMH, white matter hyperintensities; AD, Alzheimer’s disease; ATT, Arterial Transit Times; TRUST-MRI, T_2_-Relaxation-Under-Spin-Tagging Magnetic Resonance Imaging; CSF, Cerebrospinal Fluid; MCI, Mild Cognitive Impairment; VCI, vascular cognitive impairment; ATT, arterial transit time; CBF, cerebral blood flow; CMRO_2_, cerebral metabolic rate of oxygen; DSC-MRI, Dynamic Susceptibility Contrast Magnetic Resonance Imaging; MMSE, Mini-Mental State Examination.

## COMPARISON WITH ESTABLISHED AND EMERGING BIOMARKERS

OEF measurement is a relatively new way to diagnose dementia and MCI, so it is crucial to compare its merits and demerits against the current diagnostic techniques. Three primary sources are now used to analyze cognitive deterioration:

- Blood-based biological indicators, such as plasma or oxidative stress biomarkers^32^.
- Markers obtained from CSF^33^.
- PET scans that identify various biological compounds, such as fluorodeoxyglucose-PET (FDG-PET) that detects glucose metabolism, Tau-PET that detects aggregated tau (NFTs), and Amyloid PET that detects fibrillar Aβ plaques^34^.

### Plasma biomarkers

Blood biomarkers like the Aβ42/Aβ40 ratio, neurofilament light chain (NfL), glial fibrillary acidic protein, t-tau, and p-tau181 reflect neuronal degeneration-related changes^35^. Plasma NfL is the strongest cognitive decline predictor, correlating with ventricular enlargement, medial temporal atrophy, and Clinical Dementia Rating-Sum of Boxes scores. Combined with Aβ42/Aβ40 and t-tau, NfL correlates with cognitive performance on the 11-item AD Assessment Scale-Cognitive Subscale^35^. Blood biomarker tests are cost-effective and scalable versus OEF-MRI or PET, which need advanced infrastructure. Yet, these markers lack regional specificity and cannot distinguish disorders, limiting use mainly to prognosis and trial stratification^35^.

Inflammatory and oxidative stress markers like interleukin-6, TNF-α, C-reactive protein, 8-hydroxy-2′-deoxyguanosine, advanced oxidation protein products, and glutathione reflect systemic inflammation and oxidative stress early in neurodegeneration^36^. For example, 8-hydroxy-2′-deoxyguanosine indicates oxidative DNA damage, while protein oxidation and inflammation markers provide systemic snapshots. Unlike these static markers, OEF-MRI delivers a dynamic view of cerebral oxygen metabolism, localizing pathology to vulnerable regions such as watershed zones, posterior cingulate, and precuneus^37^.

### Cerebrospinal fluid biomarkers

Biomarkers such as Aβ42, t-tau, p-tau, NfL, α-Synuclein, and TAR DNA-binding protein 43 can be detected in CSF to indicate preclinical neural degeneration^38^. CSF biomarkers aid early diagnosis and differential diagnosis; for example, TAR DNA-binding protein 43 helps distinguish AD from Lewy body and frontotemporal dementias^38^. CSF analysis is more cost-effective than OEF-PET or MRI. Its temporal sensitivity and biochemical specificity support early differential diagnosis and disease monitoring^38^. However, routine clinical use is limited by procedural invasiveness, lack of biomarker standardization, and AD heterogeneity. Unlike OEF-based imaging, CSF biomarkers lack regional specificity, limiting their role compared to PET in neural degeneration treatment^37^.

### Positron emission tomography scans

PET imaging identifies affected brain regions by mapping Aβ42, t-tau, p-tau181, and fibrillar Aβ plaques (amyloid and tau PET) or assessing glucose metabolism and OEF (^15^O-PET, FDG-PET)^39^. Because PET produces static images only after sufficient target accumulation, it is mainly used for prognosis, localization, and follow-up^40^. FDG-PET consistently highlights the posterior cingulate across disorders but reveals distinct hypometabolic patterns useful for differential diagnosis. It shows high diagnostic accuracy (Area under the curve [AUC] 96.2% for dementia with Lewy bodies, 96.4% for AD, 94.7% for CN) but lower accuracy for MCI-AD (AUC 71.4%), limiting its utility for early disease^32^.

Amyloid PET is a precise tool to quantify fibrillar Aβ plaques, standardized via the centiloid scale^33^. It differentiates AD from other dementias and aids trial stratification, though plaque accumulation plateaus early, limiting prognostic value^34^. Second-generation tau PET distinguishes neurodegenerations by aggregation pattern — medial temporal and parietal cortices in AD versus frontal and anterior temporal in frontotemporal lobar degeneration^34^. Tau PET strongly correlates with cognitive impairment and disease severity, valuable for prognosis and differential diagnosis in atypical or mixed cases. Use remains limited to specialized centers and trials^34^. However, PET requires tracers, complex machinery like cyclotrons, and infrastructure; radiation exposure, need for expert interpretation, and high cost limit regular clinical use^41^.

### Oxygen extraction factor bases magnetic resonance imaging and positron emission tomography scan

QSM OEF-based MRI offers a viable alternative to PET scans, reducing radiation exposure while accurately localizing brain damage, especially in watershed zones, posterior cingulate, and precuneus. Dynamic qBOLD assesses oxygen metabolism changes longitudinally or during tasks, unlike PET’s static snapshots^41^. MRI can detect early metabolic dysfunction, identify at-risk tissue, and monitor recovery^40^. However, results may vary due to reliance on indirect biophysical models with assumptions (e.g., blood flow, hematocrit), unlike PET, which directly measures metabolism^40^. While Yang et al. (2023)^18^ do not address cost or accessibility, MRI’s noninvasive nature and widespread availability suggest QSM and qBOLD may be more accessible than PET in many clinical settings^40^. Ultimately, OEF-based MRI and PET scans can complement each other in diagnosing and treating neurodegenerative diseases.

## CLINICAL RELEVANCE AND TRANSITIONAL POTENTIAL

Cerebral OEF is a crucial physiological marker and a potential early biomarker for conditions such as stroke, AD, multiple sclerosis, sickle cell disease, and various metabolic disorders. OEF is also influenced by age and systolic blood pressure. Traditionally, OEF has been measured using PET with O-15-labeled tracers, but its clinical application is limited by the need for on-site cyclotrons, arterial blood sampling, and exposure to ionizing radiation^40^. Near-infrared spectroscopy (NIRS) has been employed in neonates to measure cerebral OEF due to sufficient light penetration in their thin skulls^10^. More recently, noninvasive MRI techniques have been developed to assess brain oxygen metabolism, offering promising applications in neurodegenerative diseases.

OEF changes in the medial temporal lobe can help detect early NVC in AD^42^. Reduced OEF, reflecting impaired neural metabolism, is characteristic of AD, whereas primary vascular pathology often results in elevated OEF as a compensatory response to reduced cerebral blood flow; reduced OEF in vascular conditions may only be seen in advanced or mixed disease states^26^. CMRO_2_, closely linked with OEF, correlates with language decline, hippocampal atrophy, and ventricular enlargement. Individuals carrying the APOE-ε4 allele with low baseline OEF or CMRO_2_ show steeper declines in executive language, and visuospatial functions, along with greater atrophy in related regions^41^. Thus, reduced baseline OEF and CMRO_2_ may accelerate cognitive and structural decline, particularly in genetically at-risk individuals^43^.

A novel algorithm, QQ-CCTV, builds on the basic QQ framework by integrating tissue-specific clustering and denoising steps to improve robustness and precision of OEF and CMRO_2_ maps^44^. Its sensitivity was shown in a caffeine challenge study, where medial temporal lobe OEF rose by 9.1% post-administration, reflecting responsiveness to physiological change^40^. Dementia remains a major global health challenge, with vascular contributions ranking second after AD. Cerebral small vessel disease (CSVD), detected via neuroimaging, includes white matter hyperintensities, lacunes, microbleeds, atrophy, enlarged perivascular spaces, and DTI-based microstructural abnormalities^45^. In acute or subacute MCA stenosis or occlusion, QSM MRI reveals elevated OEF in the affected hemisphere, a compensatory response to reduced CBF that preserves CMRO_2_ through increased oxygen extraction.

The OEF ratio, defined as the OEF in the affected hemisphere divided by that in the unaffected side, serves as a useful clinical metric. In healthy individuals, it is near 1.0, reflecting symmetric metabolism. In middle cerebral artery stroke or stenosis, the ratio often rises to ~1.2 due to compensatory extraction in hypoperfused regions. Following reperfusion or revascularization, it typically normalizes within days. For example, patients with an initial ratio of ~1.2 showed a decline to ~1.0 within three days post-intervention (P=0.03)^10^. These observations highlight OEF as a dynamic biomarker for assessing perfusion therapies and monitoring tissue viability and recovery.

**Figure 2:**
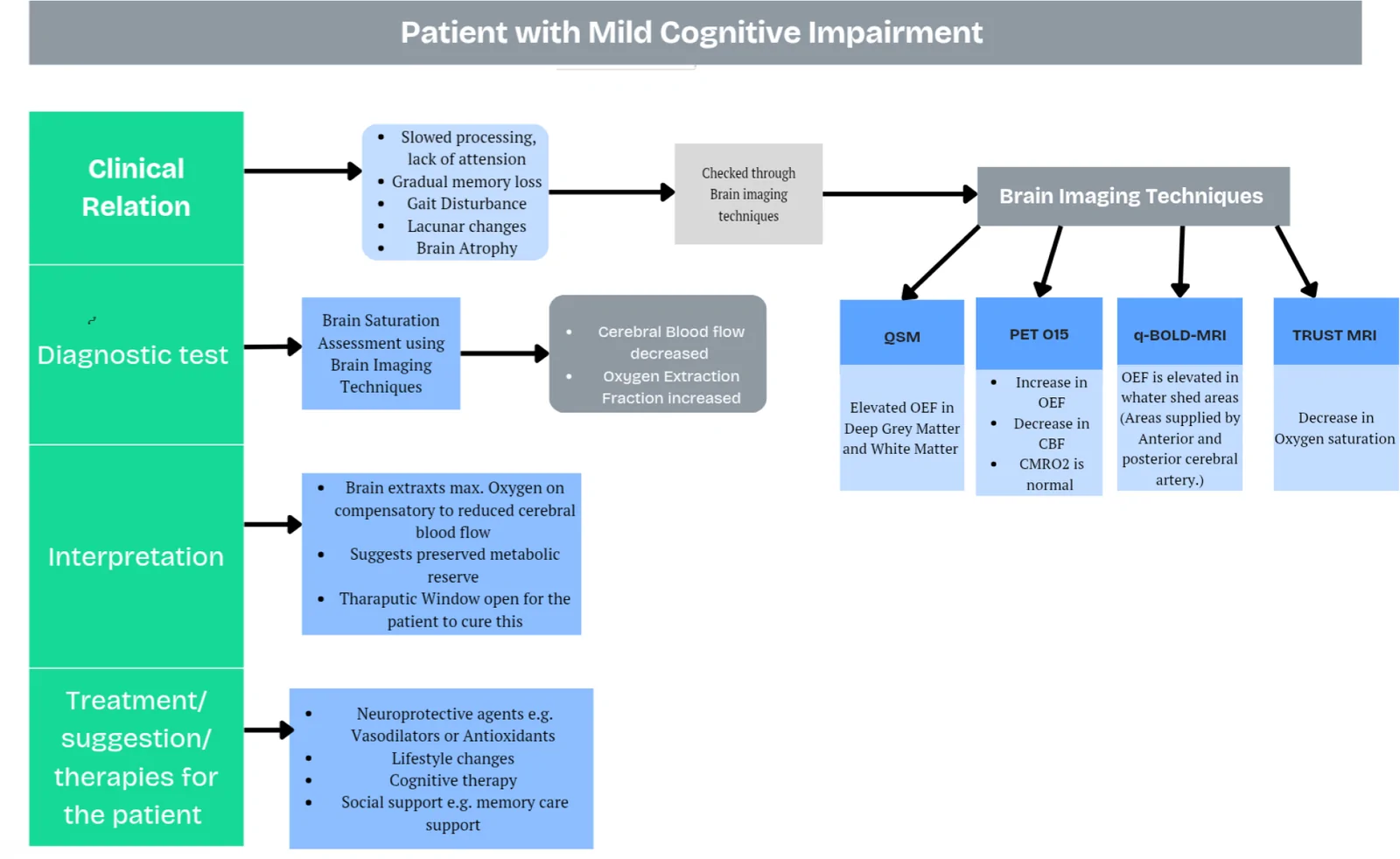
Conceptual framework linking mild cognitive impairment (MCI) with diagnostic brain imaging techniques for oxygen extraction fraction.

Abbreviations: O^15^, Positron Emission Tomography; QSM, Quantitative Susceptibility Mapping; TRUST, T^_2_^-Relaxation-Under-Spin-Tagging; MRI, Magnetic Resonance Imaging; qBOLD, Quantitative Blood Oxygen Level-Dependent.

Several therapeutic strategies may modulate OEF, including mitochondrial stabilizers (enhancing ATP production and reducing ROS), antioxidants (vitamin E, resveratrol), and anti-inflammatory agents (modulating glial activation). Cerebral vasodilators such as cilostazol improve perfusion, while cognitive enhancers like donepezil or memantine may enhance oxygen utilization at the synaptic level^46^. Figure 2 outlines the clinical manifestations of MCI, the role of brain saturation assessment and OEF-based imaging methods (QSM, PET O15, q-BOLD MRI, TRUST MRI), and subsequent interpretation leading to potential therapeutic strategies such as neuroprotective agents, lifestyle interventions, and cognitive support.

### Limitations

OEF shows promise as an AD and dementia biomarker, but faces key limitations. Most studies involve small, single-center cohorts with imbalanced case-control ratios, limiting statistical power and generalizability—for example, representative quantitative BOLD (r-qBOLD) model was applied in 30 dementia patients with 18 controls^47^, and PET-MRI vascular studies included only 24 participants^30^. TRUST MRI cohorts with 65 mixed subjects also limit robustness.

Lack of standardized protocols leads to heterogeneous methods and inconsistent results. Methods like qBOLD, PET-MRI with QSM, multi-delay arterial spin labeling (ASL), and TRUST differ in physiological assumptions, complicating comparisons. Prognostic value remains unclear; TRUST distinguishes AD from vascular impairment^28^ but does not predict outcomes. Its whole-brain estimates miss focal changes typical of early MCI.

Technical challenges include scan complexity, high costs, limited access, and operator variability^48^. Absence of reference standards restricts diagnostic use, especially given PET evidence of regional OEF variability^49^. Longitudinal data are scarce; one study showed a 0.56% greater OEF increase longitudinally over two years, while another small study showed rising OEF in AD-prone MCI regions^31^. These results may reflect an early compensatory increase in OEF predominantly driven by evolving vascular hypoperfusion rather than AD pathology, indicating that longitudinal changes in OEF are stage- and pathology-dependent. Both studies lacked clinical outcome tracking, preventing definitive conclusions about how OEF trajectories relate to cognitive or disease progression^9^.

Larger, multicenter longitudinal studies are needed to establish OEF’s utility.

### Future directions

Recent guidelines stress standardizing MRI sequence documentation for clinical and research OEF evaluations^50^. The Brain Imaging Data Structure (BIDS) is a community-driven standard that provides a consistent framework for organizing and describing neuroimaging data and associated metadata. It provides a consistent directory and file naming scheme along with structured metadata, enabling interoperable data formats that enhance transparency, sharing, and reproducibility across research groups and platforms^51^. Tools such as ezBIDS facilitate conversion of neuroimaging data into Brain Imaging Data Structure format by providing a guided interface that reduces the need for programming expertise, and enables interoperability with open repositories such as OpenNeuro.org and Brainlife.io, further supporting data sharing and collaborative analysis^52^. Adoption of dementia tools requires proving workflow feasibility and clinical value. Validated methods like volumetric MRI and plasma amyloid tests (e.g., PrecivityAD2) demonstrate scalability^53^. OEF’s adoption depends on proving its utility in prognosis, diagnosis, and management, while reducing scan time and computational demands^54^. Cost-effectiveness varies by disease, risk, and accuracy^53^.

Advanced imaging methods like spectral photon-counting and molecular imaging enhance OEF interpretation by capturing blood flow, tissue, and biochemical changes^55^. A multi-tissue, 4D approach shows spatial and temporal shifts in early neurodegeneration. Combined PET-MRI-OEF allows simultaneous assessment of structure, molecular pathology, and metabolism, improving diagnostic accuracy, distinguishing overlapping diseases, and reducing radiation exposure^56^.

Amyloid plaques detectable decades before AD symptoms via PET or CSF, with tau pathology emerging later, serve as key biomarkers^57^. OEF changes independently — rising early in vascular pathology, sometimes preceding MRI, then declining in advanced disease. This offers unique insight into cerebral oxygen metabolism, complementing other biomarkers to capture multifactorial cognitive decline^58^. OEF is promising as a therapeutic marker but requires clinical validation^40^. Imaging costs, access, and safety protocols remain challenges^59^. Limited multicenter evidence keeps it exploratory, despite biological rationale.

In conclusion, OEF remains an underutilized but promising neuroimaging biomarker for early cognitive decline and dementia. By quantifying cerebral oxygen metabolism, it reveals neurovascular and metabolic alterations preceding overt neurodegeneration. Evidence indicates OEF can aid in differentiating dementia subtypes, detecting early cerebrovascular injury, and tracking disease progression. However, limitations such as small sample sizes, limited longitudinal data, and variability among imaging methods restrict clinical translation. Future research integrating OEF with fluid biomarkers and multimodal imaging is essential to improve diagnostic accuracy. Given the clinical overlap among MCI, dementia, and aging, large-scale, rigorous studies are critical to establish OEF as a reliable clinical biomarker.

## Data Availability

No new data were generated or analyzed in this study.
